# A Laboratory Prognostic Index Model for Patients with Advanced Non-Small Cell Lung Cancer

**DOI:** 10.1371/journal.pone.0114471

**Published:** 2014-12-04

**Authors:** Arife Ulas, Fatma Paksoy Turkoz, Kamile Silay, Saadet Tokluoglu, Nilufer Avci, Berna Oksuzoglu, Necati Alkis

**Affiliations:** 1 Department of Medical Oncology, Ankara Ataturk Training and Research Hospital, Ankara, Turkey; 2 Department of Medical Oncology, Ankara Oncology Teaching and Research Hospital, Ankara, Turkey; 3 Department of Internal Medicine and Geriatrics, Yildirim Beyazit University, Faculty of Medicine, Ataturk Research and Training Hospital, Ankara, Turkey; 4 Department of Medical Oncology, Balıkesir Government Hospital, Balıkesir, Turkey; West German Cancer Center, Germany

## Abstract

**Purpose:**

We aimed to establish a laboratory prognostic index (LPI) in advanced non-small cell lung cancer (NSCLC) patients based on hematologic and biochemical parameters and to analyze the predictive value of LPI on NSCLC survival.

**Patients and Methods:**

The study retrospectively reviewed 462 patients with advanced NSCLC diagnosed between 2000 and 2010 in a single institution. We developed an LPI that included serum levels of white blood cells (WBC), lactate dehydrogenase (LDH), albumin, calcium, and alkaline phosphatase (ALP), based on the results of a Cox regression analysis. The patients were classified into 3 LPI groups as follows: LPI 0: normal; LPI 1: one abnormal laboratory finding; and LPI 2: at least 2 abnormal laboratory findings.

**Results:**

The median follow up period was 44 months; the median overall survival (OS) and median progression-free survival (PFS) were 11 and 6 months, respectively. A multivariate analysis revealed that the following could be used as independent prognostic factors: an Eastern Cooperative Oncology Group performance status score (ECOG PS) ≥2, a high LDH level, serum albumin <3 g/dL, serum calcium>10.5 g/dL, number of metastases>2, presence of liver metastases, malignant pleural effusion, or receiving chemotherapy ≥4 cycles. The 1-year OS rates according to LPI 0, LPI 1, and LPI 2 were 54%, 34%, and 17% (p<0.001), respectively and 6-month PFS rates were 44%, 27%, and 15% (p<0.001), respectively. The LPI was a significant predictor for OS (Hazard Ratio (HR): 1.41; 1.05–1.88, p<0.001) and PFS (HR: 1.48; 1.14–1.93, p<0.001).

**Conclusion:**

An LPI is an inexpensive, easily accessible and independent prognostic index for advanced NSCLC and may be helpful in making individualized treatment plans and predicting survival rates when combined with clinical parameters.

## Introduction

Lung cancer is the most common form of cancer worldwide and the leading cause of cancer-related deaths in both men and women. The prognosis for patients with advanced non-small-cell lung cancer has improved with recent advances in systemic chemotherapy and targeted therapy but still remains poor, with a median overall survival time between 4 and 15 months [1]. A substantial amount of clinical and basic scientific research has focused on the prognostic factors for patients with lung cancer. A systematic review of 887 articles revealed that there were 169 different clinical and laboratory parameters (including tumor stage, performance status, weight loss, gender, plasma lactate dehydrogenase level, and presence of bone, liver, or skin metastases) and molecular prognostic factors that have an effect on survival [2]. These molecular markers such as p53 and RAS mutations and expression of EGFR, ALK, ERCC1, beta-tubulin III, and RRM1 have been found to influence treatment outcomes [3], [4]. Testing these immunologic and histological biomarkers is not only time consuming but also their importance in standart palliative treatment is low.

New models including baseline clinical and biological factors have been developed in recent studies to predict survival in advanced cancers. Those predictive models include the Glasgow prognostic score (GPS) based on C-reactive protein (CRP) and an albumin combination, the modified Glasgow Prognostic Score (mGPS), prognostic index (PI) based on CRP and WBC, adverse prognostic factors (AFP) including 5 parameters (leukocytes>10.000 µL, ECOG>1, CA 125>35 U/mL, CYFRA 21–1>3.3 µg/L and presence of metastases), the advanced lung cancer inflammation index (ALI) based on albumin, the neutrophil-lymphocyte ratio, Montreal prognostic score (MPS) including clinical parameters, and the neutrophil-lymphocyte ratio [5]–[10]. These models might assist clinicians in making individualized treatment plans in daily practice and in planning clinical trials.

We developed a laboratory prognostic index (LPI) based on laboratory parameters that have an effect on survival by analyzing the prognostic importance of all baseline hematological, histological, and biochemical parameters and clinical characteristics of NSCLC patients. We aimed to investigate the predictive effect of this prognostic model on survival.

## Materials and Methods

### Patient Eligibility

This study was conducted at the Department of Medical Oncology in the Oncology Teaching and Research Hospital, Ankara, Turkey. The Ankara Oncology Teaching and Research Hospital Ethics Committee approved this retrospective study in May, 2009. Patients’ records and information were anonymized and de-identified prior to analysis. The investigation was a retrospective and single center study. These patients were treated and received follow-up evaluations between June, 2000 and April, 2010 in our hospital. A total of 1,320 NSCLC patients were reviewed. The inclusion criteria were: 1) patients were histologically or cytologically diagnosed as primary NSCLC and staged according to the tumor-node-metastasis (TNM) criteria [11] and 2) patients were diagnosed as stage IIIB and IV of their disease. Patients were excluded if they 1) were SCLC or did not have a primary diagnosis of lung cancer; 2) were stage I, II and IIIA; 3) could not provide detailed clinical data; 4) had missing laboratory data (i.e., WBC, hemoglobin, platelet, Alkaline phosphatase, Lactate dehydrogenase, albumin, or calcium); 5) underwent surgery; 6) had clinical evidence of active infection or inflammation; 7) had hematological disease 8) had had a pulmonary embolism, acute myocardial infarction, or cerebrovascular accident within one month. After exclusion, 462 cases were found eligible for analysis.

### Clinical and Laboratory Data Collection

The data included demographic information, histological classification, clinical staging, hematological information including white blood cell (WBC) count, hemoglobin (Hgb) level, platelet (PLT) count, and biochemical parameters such as albumin, serum calcium level, lactate dehydrogenase (LDH), and alkaline phosphatase (ALP) levels. The cut-off value for each biological baseline parameter was defined as follows: anemia: a hemoglobin level of less than 11 g/dL; leukocytosis: a white blood cell count over 10.000/µL; thrombocytosis: a platelet count over 450.000/µL; hypoalbuminemia: a serum albumin level of less than 3.0 g/dL; alkaline phosphatase level and lactate dehydrogenase level: above normal level, according to standard laboratory norms; and serum calcium level: hypercalcemia >10.5 gr/dL.

The patients’ performance statuses were recorded according to the Eastern Cooperative Oncology Group performance status scores. The sites of distant metastases were recorded. The initial treatment modalities included chemotherapy (CT) and palliative radiotherapy. The patients who did not receive any anti-tumor treatment were defined as receiving the best supportive care (BSC). Data on the type of CT regimen and number of CT cycles were collected. Therapeutic responses were evaluated using criteria determined by the World Health Organization [12]. A complete response was defined as an inability to detect a disease-related lesion; a partial response (PR) was defined as the shrinking of the lesion by at least 50%; a stable disease (SD) was defined as the shrinking of the lesion by less than 50% or the enlargement of the lesion by up to <25%; and a progressive disease (PD) was defined as the enlargement of the lesion by>25% or the formation of new lesions.

Overall survival (OS) was calculated from the diagnosis of the patient to either the date of death from any cause or the date of the last follow-up. Progression-free survival (PFS) was calculated as the interval between the beginning of treatment and the progression of the disease, recurrence, or death from any cause. All survival data were updated in August, 2011.

### Statistical Analysis

The Pearson Chi-square test for frequencies and *analysis of variance* (ANOVA) for means was used to compare clinicopathological and laboratory parameters among groups. A Kaplan-Meier analysis with log-rank test was used to determine cumulative survival curves.

The factors included in a univariate Cox proportional hazards regression model with 95% confidence intervals (95% CI) for PFS and OS were age (≤65 vs.>65); gender (male vs. female); ECOG PS (≥2 vs. 0–1); histology (squamous vs. nonsquamous including adenocarcinoma, large cell, and unspecified NSCLC histology); weight loss (<5% vs. ≥5% in the last 6 months); history of smoking (yes vs. no); site of metastases (presence vs. absence of bone, brain, liver, contralateral lung, adrenal gland); number of metastatic sites (no, 1–≤2 vs.>2); palliative chemotherapy (no, 1–3 cycles vs. ≥4 cycles); as well as laboratory parameters such as WBC (leucocytosis vs. normal); hemoglobin level (anemia vs. normal); PLT count (trombocytosis vs. normal); LDH (high vs. normal); albumin (hypoalbuminemia vs. normal); ALP (high vs. normal); and calcium (hypercalcemia vs. normal). In order to select those factors with independent significant influence on OS and PFS, multivariate analyses were carried out in a backward stepwise Cox’s proportional hazards model. We developed an LPI the included serum levels of WBC, LDH, albumin, calcium and ALP based on the results of the Cox regression analysis.

Statistical analyses were performed using SPSS 15.0 software (SPSS Inc. Chicago, IL). All statistical assessments were two-sided and a p-value of <0.05 was considered statistically significant.

## Results

### Patient Characteristics

Our study included a total of 462 advanced patients with NSCLC including 405 males and 57 females. The median age was 58 years (range: 22 to 85 years) and 76.6% of the patients were younger than 65 years. Seventy-six percent of the patients had a history of smoking (median 45 pack/year, range: 10–180 pack/year) and 58.2% had non-squamous carcinoma. Forty-five percent of the patients had stage IIIB and 54.6% were stage IV. The most common site of metastases was the brain (16.2%), followed by the liver (10.2%), surrenal (10.2%), and bone (8.4%).

The median and minimum-maximum number of baseline hematological and biochemical parameters were recorded as: WBC 9.6×10^3^/µL (1.4–66.7), Hgb 12.8 g/dL (6.50–20.0), PLT 338.5×10^3^/µL (94.5–1150.0), LDH 335.5 U/L (67–3760), ALP 140 U/L (90–2168), Calcium 9.2 mg/dL (8.0–15.7), and albumin 3.4 g/dL (1.60–4.8).

Of the patients who were treated with chemotherapy, 393 received first-line platinum-doublet therapy and 159 received second-line palliative therapy. Due to poor performance status or comorbidities, 14.9% of the patients received BSC only. The most common first-line regimens were cisplatin/gemcitabine (n = 154), cisplatin/etoposide (n = 111), cisplatin/vinorelbine (n = 67), cisplatin/docetaxel (n = 40), and carboplatin/vinorelbine (n = 20). The median number of chemotherapy cycles was 4 (range: 2 to 7) among patients who received chemotherapy as a first line regimen. The overall response rate was 43.5% (including 10.8% stable disease and 32.7% partial response). One hundred eight-one patients received radiation therapy with concurrent chemotherapy.

### Survival and Prognostic Factors

The median follow-up duration was recorded as 44 months (3–134). During that time, 438 patients (94.8%) progressed and 391 (84.6%) patients died. Median OS was 11 months (95% CI; 9.85–12.15) and median PFS was 6 months (95% CI; 5.42–6.58). The actual 1-, 2- and 5-year OS rates were 42.4%, 19.3%, and 5.8%, respectively. The baseline characteristics of patients and their survival rates are shown in Table 1.

**Table 1:** Baseline Characteristics, Overall Survival and Progression-Free Survival of Patients.

Characteristics	Number of Patients (%)	6-month PFS (%)	P_logrank_	1-year OS (%)	P_logrank_
**Age at diagnosis**			**0.851**		**0.092**
<65 years	354 (76.6)	25		32	
≥ 65 years	108 (23.4)	27		27	
**Sex**			**0.530**		**0.475**
Male	405 (87.7)	26		30	
Female	57 (12.3)	21		35	
**Histopathology**			**0.099**		**0.822**
Squamous	181 (39.2)	26		29	
Non-squamous	269 (58.2)	24		32	
Other	12 (2.6)	25		24	
**ECOG PS**			**<0.0001**		**<0.0001**
ECOG <2	219 (47.4)	32		44	
ECOG ≥2	243 (52.6)	19		19	
**History of smoking**			**0.128**		**0.504**
No	70 (15.2)	27		35	
Yes	352 (76.2)	24		30	
Unknown	40 (8.7)	30		25	
**Weight loss**			**0.859**		**0.064**
<5%	164 (35.9)	27		28	
≥5%	214 (46)	24		23	
Unknown	84 (18.1)	26		24	
**WBC**			**<0.0001**		**<0.0001**
Normal	253 (54.8)	31		36	
High (≥10.000/uL)	209 (45.2)	18		23	
**Hemoglobin**			**0.086**		**0.195**
Normal	307 (66.5)	28		31	
Anemia (<12 g/dl)	155 (33.5)	20		30	
**Platelet**			**0.339**		**0.240**
Normal	346 (74.9)	27		31	
High (> 450.000/uL)	116 (25.1)	21		28	
**LDH (U/L)**			**<0.0001**		**<0.0001**
Normal	196 (42.4)	33		40	
High	266 (57.6)	19		23	
**ALP**			**<0.0001**		**0.001**
Normal	383 (82.9)	28		33	
High	79 (17.1)	11		19	
**Calcium**			**<0.0001**		**<0.0001**
Normal	397 (85.9)	27		34	
High (>10.5 g/dl)	65 (14.1)	10		9	
**Albumin**			**0.002**		**<0.0001**
Normal	371 (80.3)	28		35	
Low (<3 g/dl)	91 (19.7)	16		14	
**Number of metastasis**			**<0.0001**		**<0.0001**
0	210 (45.5)	37		41	
1- ≤2	218 (47.2)	17		24	
>2	34 (7.4)	6		7	
**Liver metastasis**			**<0.0001**		**<0.0001**
No	416 (89.8)	25		31	
Yes	46 (10.2)	4		5	
**Surrenal metastasis**			0.027		0.008
No	416 (89.8)	23		29	
Yes	46 (10.2)	14		18	
**Brain metastasis**			0.002		**<0.0001**
No	388 (83.8)	24		30	
Yes	74 (16.2)	12		15	
**Bone metastasis**			**<0.0001**		**<0.0001**
No	423 (91.6)	27		30	
Yes	39 (8.4)	9		15	
**Malign pleural effusion**					
No	423 (91.6)	24	0.024	29	0.003
Yes	39 (8.4)	7		11	
**Contralateral lung**			0.772		0.155
No	421 (91.1)	33		40	
Yes	41 (8.9)	21		27	
**Chemotherapy**			**<0.0001**		**<0.0001**
≥4 cycles	156 (33.8)	41		50	
1-3 cycles	237 (51.3)	9		12	
No	69 (14.9)	5		4	

*ECOG* Eastern Cooperative Oncology Group, *PS* performance status, *Nonsquamous histology* including adenocarcinoma, large cell, and unspecified NSCLC histology, *WBC* White Blood Cell count, *LDH* Lactate dehydrogenase, *ALP* Alkaline phosphatase.

A univariate analysis revealed the following parameters were effective for PFS: ECOG PS≥2 (p = 0.002), WBC≥10.000 (p = 0.028), high LDH level (p = 0.045), high ALP level (p = 0.015), hypercalcemia (p = 0.002), hemoglobin <10 gr/dL (p = 0.039), number of metastases>2 (p<0.0001), presence of pleural effusion (p = 0.001), presence of liver metastases (p<0.0001), presence of brain metastases (p<0.0001) and presence of bone metastases (p<0.0001), number of chemotherapy cycles ≥4 (p<0.0001). A multivariate analysis showed that a high level of LDH (HR: 1.36, 1.07–1.73, p = 0.011), a number of metastases>2 (HR: 1.76, 1.06–2.94, p = 0.003), and a number of chemotherapy cycles ≥4 (HR: 0.25, 0.19–0.32, p<0.0001) were independent prognostic factors related to PFS in advanced NSCLC patients.

The parameters which were found by univariate analysis to have the greatest effect on overall survival were ECOG PS≥2, WBC≥10.000, high LDH level, high ALP level, hypercalcemia, albumin <3 gr/dL, number of metastases>2, presence of pleural effusion, brain, liver, surrenal and bone metastases and number of chemotherapy cycles ≥4. A multivariate analysis showed that ECOG PS≥2 (HR: 1.33, 1.06–1.66, p = 0.014), high LDH level (HR: 1.31, 1.00–1.70, p = 0.047), serum albumin <3 g/dL (HR: 1.28, 0.98–1.67, p = 0,037), serum calcium>10.5 g/dL (HR: 1.46, 1.09–1.96, p = 0.011), metastases number>2 (HR: 2.11, 1.42–3.14, p<0.0001), presence of metastases (HR: 1.73, 1.11–2.71, p = 0.016), presence of pleural effusion (HR: 1.98, 1.33–2.94, p = 0.001), and a number of chemotherapy cycles ≥4 (HR: 0.53, 0.38–0.73, p<0.0001) were found to be related to OS as independent prognostic factors. The significant variables for OS in univariate and multivariate analyses are summarized in Table 2.

**Table 2:** Results of univariate and multivariate Cox's proportional hazard models regarding OS.

Characteristics	Univariate Analysis		Multivariate Analysis	
	OS HR (95% CI)	P value	OS HR (95% CI)	P-value
**Age (>65 vs. ≤65 years)**	1.20 (0.95-1.51)	0.130	-	-
**Sex (Male vs. Female)**	1.18 (0.86-1.63)	0.303	-	-
**Histology (Squamous vs. Nonsquamous)**	1.02 (0.83-1.25)	0.850	-	-
**History of smoking (Yes vs. No)**	1.16 (0.87-1.55)	0.309	-	-
**Surrenal metastasis (Yes vs. No)**	1.33 (0.99-1.71)	0.058	-	-
**Brain metastasis (Yes vs. No)**	1.56 (1.26-1.98)	<0.0001	-	-
**Bone metastasis (Yes vs. No)**	1.67 (1.38-2.01)	<0.0001	-	-
**Contralateral lung (Yes vs. No)**	1.23 (0.89-1.69)	0.211	-	-
**Hemoglobin (Low vs. Normal)**	1.13 (0.92-1.39)	0.260	-	-
**Platelets (High vs. Normal)**	1.11 (0.88-1.39)	0.387	-	-
**ALP (High vs. Normal)**	1.51 (1.16-1.97)	0.002	-	-
**WBC (High vs. Normal)**	1.55 (1.27-1.89)	<0.0001	-	-
**LDH (High vs. Normal)**	1.45 (1.18-1.77)	<0.0001	1.31 (1.00-1.70)	0.047
**Albumin (Low vs. Normal)**	1.81 (1.42-2.29)	<0.0001	1.28 (0.98-1.67)	0.037
**ECOG PS (<2 vs. ≥2)**	1.99 (1.63-2.44)	<0.0001	1.33 (1.06-1.66)	0.014
**Calcium (High vs. Normal)**	2.36 (1.79-3.11)	<0.0001	1.46 (1.09-1.96)	0.011
**Liver metastasis (Yes vs. No)**	2.31 (1.77-3.00)	<0.0001	1.73 (1.11-2.71)	0.016
**Malign pleural effusion (Yes vs. No)**	1.66 (1.23-1.98)	0.001	1.98 (1.33-2.94)	0.001
**Chemotherapy**		<0.0001		<0.0001
No vs. 1-3 cycles	0.48 (0.36-0.64)		0.53 (0.38-0.73)	
No vs. ≥4 cycles	0.14 (0.11-0.19)		0.16 (0.11-0.22)	
**Number of metastasis**		<0.0001		<0.0001
0 vs. 1- ≤2	1.59 (1.29-1.97)		1.53 (1.23-1.90)	
0 vs.>2	2.73 (1.86-4.01)		2.11 (1.42-3.14)	
**LPI**		<0.0001		<0.0001
0 vs. 1	1.49 (1.11-1.98)		1.41 (1.05-1.88)	
0 vs. ≥2	2.52 (1.92-3.31)		1.99 (1.46-2.70)	

*OS* overall survival, *HR* hazard ratio, *CI* confidence interval, *LPI* laboratory prognostic index, *ECOG* Eastern Cooperative Oncology Group, *PS* performance status, *WBC* White Blood Cell count, *LDH Lactate dehydrogenase, ALP* Alkaline phosphatase.

### Laboratory Prognostic Index (LPI)

LPI was developed based on laboratory parameters (WBC, LDH, ALP, Calcium, Albumin) that were found to have an effect on survival. Hemoglobin and platelet levels were ignored since their levels were found to be statistically insignificant (P_logrank_ = 0.195 and P_logrank_ = 0.240, respectively). These 5 parameters were scored as “0” if their level was in normal range and “+1” if their level was abnormal.

All laboratory parameters were normal in 20.6% of patients (n = 95). In 153 patients (33.1%), only one parameter was normal; in 113 patients (24.5%), two parameters were normal; in 78 patients (16.9%), three parameters were normal; and in 18 patients (3.9%), four parameters were abnormal. All laboratory parameters were abnormal in only 5 patients (1.1%).

Patients were classified into 3 LPI groups as follows: LPI 0: all laboratory parameters (WBC, LDH, ALP, Ca, Albumin) were normal; LPI 1: one laboratory parameter was abnormal; and LPI 2: at least two laboratory parameters were abnormal. The number of patients in groups LPI 0, 1, and 2 were 95, 153, and 214, respectively.

**Figure 1:**
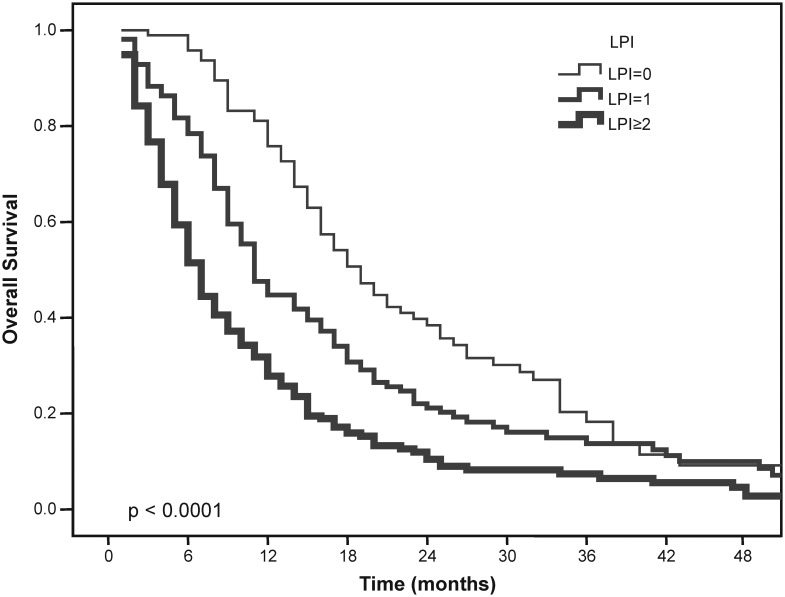
Overall survival of patients according to LPI.

**Figure 2:**
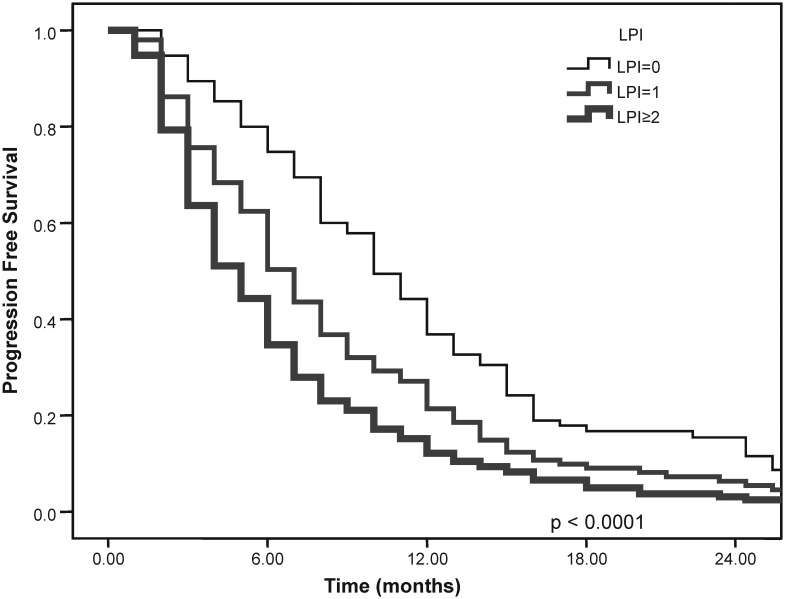
Progression-free survival of patients according to LPI.

The overall survival of patients based on their LPI groups was as follows: 19 months (15.8–22.2) in patients with an LPI score of 0; 11 months (9.5–12.5) in patients with an LPI score of 1; and 7 months (6.1–7.9) in patients with an LPI score of 2 (P_log rank_<0.0001). The median PFS was found to be 10 months (8.5–11.5) in patients with an LPI score of 0; 7 months (6.2–7.8) in patients with an LPI score of 1; and 5 months (4.3–5.7) in patients with an LPI score ≥2 (P_log rank_<0.0001). The OS and PFS curves of patients according to LPI are shown in Figures 1 and 2.

The demographic and clinical parameters of patients according to LPI groups and 1-year OS rates are shown in Table 3. There were no differences in gender, age, histopathology, and history of smoking among LPI groups. Although the patients with a high LPI score had poorer PS, multiple metastases, and lower chemotherapy receiving rates, the impact of LPI on survival was independent of those variables.

**Table 3:** Overall Survival according to patient characteristics and LPI.

Characteristics	Number of Patients (%)			P-value	1-year Overall Survival (%)			P-value
	LPI = 0 (n = 95)	LPI = 1 (n = 153)	LPI ≥ 2 (n = 214)		LPI = 0 (n = 95)	LPI = 1 (n = 153)	LPI ≥ 2 (n = 214)	
**ECOG PS**				<0.0001				
ECOG <2	61 (64.2)	76 (49.7)	82 (38.3)		58	50	26	<0.0001
ECOG ≥2	34 (35.8)	77 (50.3)	132 (61.7)		47	19	22	<0.0001
**No of metastasis**				<0.0001				
0	45 (47.4)	87 (56.9)	78 (36.4)		66	40	27	<0.0001
1- ≤2	48 (50.5)	59 (38.6)	111 (51.9)		45	28	14	<0.0001
>2	2 (2.1)	7 (4.6)	25 (11.7)		19	4	0	0.009
**Chemotherapy**				<0.0001				
>3 cycles	67 (70.5)	84 (54.9)	86 (40.2)		63	51	39	0.003
1-3 cycles	25 (26.3)	47 (30.7)	84 (39.3)		32	15	3	<0.0001
No	3 (3.2)	22 (14.4)	44 (20.6)		33	9	0	<0.0001
**Age at diagnosis**				0.862				
<65 years	71 (74.7)	117 (76.5)	166 (77.6)		51	38	19	<0.0001
≥65 years	24 (25.3)	36 (23.5)	48 (22.4)		63	22	11	<0.0001
**Sex**				0.446				
Male	81 (85.3)	132 (86.3)	192 (89.7)		52	35	17	<0.0001
Female	14 (14.7)	21 (13.7)	22 (10.3)		63	28	21	0.011
**Histopathology**				0.443				
Squamous	31 (33.0)	72 (48.0)	78 (37.9)		47	38	14	<0.0001
Non-squamous	63 (67.0)	78 (52.0)	128 (62.1)		57	31	20	<0.0001
**Smoking history**				0.978				
No	15 (17.2)	24 (16.7)	31 (16.2)		59	36	21	0.018
Yes	72 (82.8)	120 (83.3)	160 (83.8)		52	35	17	<0.0001

*LPI* laboratory prognostic index, *ECOG* Eastern Cooperative Oncology Group, *PS* performance status.

The relationship between treatment arms and LPI score was found to be statistically significant. For patients with a LPI score of 0; the median OS were 27 (17.5–36.4) and 16 (13.7–18.2) months in patients receiving chemoradiotherapy and palliative chemotherapy, respectively (P_logrank_ = 0.002). On the other hand, the median OS were 9 (5.8–12.1) and 6 (5–7) months in patients with a LPI score >2 who received chemoradiotherapy and palliative chemotherapy, respectively (P_logrank_ = 0.011).

The mortality risk was higher with an HR of 1.49 (95% CI 1.11–1.98) for patients with one abnormal laboratory parameter and an HR of 2.52 (95% CI 1.2–3.31) for patients with ≥2 abnormal laboratory parameters, compared to those patients with normal laboratory parameters (P<0.0001). The risk of progression was significantly higher for patients with ≥2 abnormal laboratory parameters (HR: 2.02; 95% CI 1.57–2.60) and with one abnormal laboratory finding (HR: 1.48; 95% CI 1.14–1.93), compared to patients with normal parameters (P<0.0001). A multivariate Cox’s regression analysis identified that an LPI was an independent prognostic factor for NSCLC (P<0.0001).

## Discussion

In this study, we developed a practical index based on laboratory parameters and demonstrated its predictive effect on the survival of advanced NSCLC patients. We analyzed 21 clinical, hematological, and biochemical factors. A laboratory prognostic index that was established based on five laboratory parameters and which was found to be statistically significant with a Cox regression analysis (WBC, LDH, ALP, Calcium, and Albumin) can be used as a predictor for both OS and PFS. A multivariate analysis revealed that the mortality and progression risks in patients with an LPI score of 1 increases by 1.41 and 1.48 times, respectively.

NSCLC is a heterogeneous disease that can have extreme differences in prognosis. The differences among the tumor biology and behavior may contribute to this heterogeneity. In the last few decades, researchers developed nomogram and survival indexes by investigating clinical factors in order to predict the individual prognoses of NSCLC patients. The first analysis, which was completed in the mid-1980s based on two ECOG randomized phase III studies data, developed a mathematical description model to predict the survival of NSCLC patients. Eight factors having a positive effect on survival were detected: PS 0, female sex, no bone metastasis, no weight loss, no subcutaneous metastasis, non-large cell histology, no prior symptoms of shoulder or arm pain, and no liver metastasis [13]. The second largest analysis, published in 1991, revealed that four pretreatment factors were positively associated with survival. These factors were PS 0 to 1, cisplatin based therapy, female sex, and age ≥70 years [14]. The third largest analysis, published in 1995, found eight factors that have a negative effect on survival: Karnofsky PS 70, leukocytosis, skin metastasis, increased calcium level, abnormal neutrophil count, age >60 years, and male gender [15]. In the mid-2000s, Hoang and et al. published another study revealing six independent prognostic factors in patients who were treated with third generation platinum based doublets. These factors included skin metastasis, ECOG PS 1 or 2, loss of appetite, liver metastasis, metastases ≥4, and no prior surgery. Hematological parameters were not included in the study [16].

In our study, age, histology, and smoking history were found to be irrelevant in relation to survival. Some previous studies reported that being 70 years and older was a positive prognostic factor for NSCLC but others found no significant association between age and prognosis [14], [16]. On the other hand, survival in younger patients was better in some studies [17], [18].

Survival in younger patients was better in some studies [17], [18]. Although the incidence of NSCLC in women has increased due to more women smoking in the last half of the 20^th^ century, in our population, the ratio of women patients was 12.8%. In light of recent studies, it known that there are gender differences in the occurrence of lung cancer. Women are more susceptible to the carcinogenic effects of cigarettes and have more adenocarcinoma and EGFR mutations [19]. Hirsh et al. reviewed 11 articles and found a relationship between histology and prognosis. While some studies showed adenocarcinoma and nonsquamous histologies as positive prognostic factors, the others showed squamous cell carcinoma histology as a positive prognostic factor [20]. In our study, squamous cell cancer was the most common histological type. This might be due to the long follow-up period and high unspecified NSCLC rate (21.8%) of our population. Among the LPI groups, there were no differences between age, sex, histology, and smoking history. The effect of an LPI score on survival was independent of these factors.

In large studies including lung cancer patients, PS was found as the most important negative prognostic factor [13]–[16]. In our study, PS was also found to be an independent prognostic factor. In patients with a poor ECOG PS, a 1-year OS rate was higher in the LPI 0 group than in the LPI 2 group. In previous studies, the Glasgow Prognostic Score (GPS) was found to be superior to the ECOG PS to predict prognosis [5]; however, 3 to 6 months after diagnosis, neither the GPS nor the ECOG PS was found to be superior, which was most likely due to the deterioration of the ECOG PS and GPS over time [21].

Unlike the data on performance status, there is conflicting data on the impact of weight loss on survival. In our study, we found that weight loss had no significant effect on survival (which is consistent with some of the previously published data), whereas some studies showed an association between poor survival and weight loss [13]–[16], [22].

In our study, the most common sites of metastases were the brain and liver. The presence of liver metastases (HR: 1.73) and the presence of malignant pleural effusion (HR: 1.98) were detected as important independent prognostic markers. The presence of hepatic metastases was found to be associated with short survival time in most of the studies [13], [16]. Malignant pleural effusion was evaluated as metastatic disease and M1a in TNM staging [23]. The number of patients with metastatic disease was higher in the LPI high score group. The LPI score was shown to predict survival in each group regardless of the number of metastases.

In cancer patients, leukocytosis and poor performance status were found to be associated with short survival time [15], [24]–[26]. Kasymjanova et al. defined a PI based on the pretreatment inflammatory markers CRP and WBC in advanced NSCLC. The median OS rates were 20 months for the PI 0 group, 10.4 months for the PI 1 group, and 7.9 months for the PI 2 group (p<0.001) [7]. Gagnon et al. developed an MPS based on LDH, albumin, CRP, and neutrophil lymphocyte ratio in incurable lung cancer patients. The median OS rates were 2.5, 8.2 and 18.2 months for groups 1, 2, and 3, respectively [10]. In our scoring system, the median survival rates were 19, 11, and 7 months for LPI scores of 0, 1, and 2, respectively. Survival curves were similar to Gagnon et al.’s study.

In our study, a high serum LDH level was associated with short-term survival. LDH, which is related to intratumoral hypoxia, increases macrophage mediated angiogenesis and invasion ability [14], [26]–[28]. An increased LDH level was shown to be associated with chemotherapy and radiation therapy resistance [29]. An elevated level of LDH-5 in tumor tissue samples was found to be related to some angiogenic factors’ (VEGF, bFGF, bFGFR) expression and shown to be a poor prognostic factor for NSCLC patients [30]. Further prospective studies are needed in order to determine the clinical use of tumor LDH expression.

Hypoalbunemia is linked with malnutrition and is very common in lung cancer patients. Malnutrition is also related to low quality of life, decreases in response to treatment, increases in toxicity risk (which is induced with chemotherapy), and decreases in survival rate [15], [26], [31], [32]. In our study, a low serum albumin level was a poor prognostic factor with an HR of 1.28. Some scoring systems were developed based on CRP and albumin. In the GPS scoring system, which included these markers, the survival times were 11.6, 8.4 and 1.2 months for GPS 0, 1, and 2, respectively [21]. The other scoring systems (mGPS, ALI, and MPS) also revealed that hypoalbunemia was associated with poor survival [6], [9], [10].

Hypercalcemia may occur in advanced stages of NSCLC and is a poor prognostic factor [14], [15], [33]. In cancer patients, many mechanisms play a role in hypercalcemia development and can be seen either as a paraneoplastic syndrome or as a result of bone metastases [34]. The most common mechanism is the secretion of a parathyroid hormone–related peptide by tumor cells [35]. In our study, hypercalcemia was found to be a poor prognostic factor.

Most of our patients were treated with third generation platinum-based doublets. While the 1-year OS rate was 4% in patients who did not receive any chemotherapy, it was 12% in patients who received 1–3 cycles of chemotherapy and 50% in patients who received ≥4 cycles of palliative CT. A meta-analysis demonstrated that cisplatin-based chemotherapy improved survival rate, quality of life, and performance status, compared with BSC [36].

In our study, the 1-year survival rates of patients who were treated with at least 4 cycles of CT were 63%, 51%, 39% in LPI 0, LPI 1 and LPI 2 groups, respectively. The 1-year survival rates according to LPI scores of 0, 1, and 2 were 33%, 9%, and 0% in patients who did not receive any CT.

Trape et al. developed an AFP scoring system that revealed similar results to our study. In their study, the median OS rates of patients who received CT were 15, 7, and 5 months in AFP 0–1, AFP 2–3 and AFP 4–5 groups, respectively, versus 8, 6, and 2 months in patients who did not receive any CT [8]. The median PFS was 10 months in the LPI 0 group and 5 months in the LPI 2 group. We considered evaluating LPI groups according to chemotherapy regimens but the results would be statistically insignificant due to low patient number and heterogeneity of chemotherapeutic agents. Kasymjanova et al. showed that a PI was a valuable predictor both at diagnosis and after 2 cycles of chemotherapy in advanced stage patients (p = 0.007) [7].

We believe that future prospective clinical trials using the LPI score would help clinicians determine which group of patients may benefit from standard chemotherapy versus palliative care. This simple index would help clinicians determine the high risk patients and improve the patients’ quality of life by planning palliative care.

In previous studies, laboratory parameters were rarely evaluated, due to difficulties with confirming their accuracy since they were conducted at different centers. Our patient population, however, belonged to a single cancer center and had the same reference range for laboratory parameters.

Our limitations were as follows: the fact that our study included a heterogeneous group, since there were no restrictions according to age, PS, metastases sites, or treatment. The relationship between LPI scores and chemotherapy regimens could not be analyzed statistically, since different chemotherapy regimens were included in the study. Unfortunately, we could not analyze the correlation of targeted therapies such as crizotinib and erlotinib with LPI scores since these therapies were unavailable prior to 2010.

The other limitation was that the baseline parameters upon which LPI was based might change over time. The continuity of the LPI’s predictive value should be evaluated by prospective studies. For more accurate results, the LPI might be calculated periodically (e.g., every 3 to 6 months).

In conclusion, we have developed an inexpensive, easily accessible, and independent prognostic index for advanced NSCLC patients. Even though immunologic and histological markers were identified, we could not obtain most of these factors in our daily practice. We believe that in the future, cancer treatment will be completely individualized in association with increased genetic profiling. LPI might be complementary to prognostic models based on genetic profiles. We think this index will assist us in making individualized treatment plans, deciding which patient groups would benefit from chemotherapy, and planning clinical studies in advanced NSCLC patients.

## References

[1] Hotta K, Fujiwara Y, Kiura K, Takigawa N, Tabata M, et al (2007) Relationship between response and survival in more than 50,000 patients with advanced non-small cell lung cancer treated with systemic chemotherapy in 143 phase III trials. J Thoracic Oncol 2:402–7. 10.1097/01.JTO.0000268673.95119.c7 17473655

[2] Brundage MD, Davies D, Mackillop WJ (2002) Prognostic factors in non-small cell lung cancer: a decade of progress. Chest 122:1037–1057. 12226051 10.1378/chest.122.3.1037

[3] Rosell R, Fossella F, Milas L (2002) Molecular markers and targeted therapy with novel agents: prospects in the treatment of non-small cell lung cancer. Lung Cancer 38 Suppl 4: 43–49. 10.1016/s0169-5002(02)00171-x 12480194

[4] Kulesza P, Ramchandran K, Patel JD (2011) Emerging concepts in the pathology and molecular biology of advanced non-small cell lung cancer. Am J Clin Pathol 136:228–38. 21757595 10.1309/AJCPO66OIRULFNLZ

[5] Forrest LM, McMillan DC, McArdle CS, Angerson WJ, Dunlop DJ (2003) Evaluation of cumulative prognostic scores based on the systemic inflammatory response in patients with inoperable non-small-cell lung cancer. Br J Cancer 89:1028–1030. 12966420 10.1038/sj.bjc.6601242 PMC2376960

[6] Leung EY, Scott HR, McMillan DC (2012) Clinical utility of the pretreatment Glasgow prognostic score in patients with advanced inoperable non-small cell lung cancer. J Thorac Oncol 7(4):655–662. 22425914 10.1097/JTO.0b013e318244ffe1

[7] Kasymjanova G, MacDonald N, Agulnik JS, Cohen V, Pepe C, et al (2010) The predictive value of pre-treatment inflammatory markers in advanced non-small-cell lung cancer. Curr Oncol 17(4):52–58. 10.3747/co.v17i4.567 PMC2913830 20697515

[8] Trape J, Montesinos J, Catot S, Buxo J, Franquesa J, et al (2012) A prognostic score based on clinical factors and biomarkers for advanced non-small cell lung cancer. Int J Biol Markers 27(3):257–62. 10.5301/JBM.2012.9314 22815214

[9] Jafri SH, Shi R, Mills G (2013) Advance lung cancer inflammation index (ALI) at diagnosis is a prognostic marker in patients with metastatic non-small cell lung cancer (NSCLC): a retrospective review. BMC Cancer 13:158–167. 23530866 10.1186/1471-2407-13-158 PMC3618002

[10] Gagnon B, Agulnik JS, Gioulbasanis I, Kasymjanova G, Morris D, et al (2013) Montreal prognostic score: estimating survival of patients with non-small cell lung cancer using clinical biomarkers. British Journal of Cancer 109:2066–71. 24064979 10.1038/bjc.2013.515 PMC3798950

[11] Vallieres E, Shepherd FA, Crowley J, Van Houtte P, Postmus PE, et al (2009) The IASLC Lung Cancer Staging Project: proposals regarding the relevance of TNM in the pathologic staging of small cell lung cancer in the forthcoming (seventh) edition of the TNM classification for lung cancer. J Thorac Oncol 4(9):1049–59. 19652623 10.1097/JTO.0b013e3181b27799

[12] Travis WD, Brambilla E, Muller-Hermlink HK, Harris CCeditors. (2004) World Health Organization classification of tumours. Pathology and genetics of tumours of the lung, pleura, thymus and heart. Lyon: IARC Press.

[13] Finkelstein DM, Ettinger DS, Ruckdeschel JC (1986) Long-term survivors in metastatic non-small cell lung cancer: An Eastern Cooperative Oncology Group Study. J Clin Oncol 4:702–709. 3701389 10.1200/JCO.1986.4.5.702

[14] Albain KS, Crowley JJ, LeBlanc M, Livingston RB (1991) Survival determinants in extensive-stage non-small cell lung cancer: The Southwest Oncology Group experience. J Clin Oncol vol 9(9):1618–1626. 10.1200/JCO.1991.9.9.1618 1651993

[15] Paesmans M, Sculier JP, Libert P, Bureau G, Dabouis G, et al (1995) Prognostic factors for survival in advanced non-small cell lung cancer: univariate and multivariate analyses including recursive partitioning and amalgamation algorithms in 1,052 patients. The European Lung Cancer Working Party. J Clin Oncol 13:1221–1230. 7738625 10.1200/JCO.1995.13.5.1221

[16] Hoang T, Xu R, Schiller JH, Bonomi P, Johnson DH (2005) Clinical model to predict survival in chemo naive patients with advanced non-small-cell lung cancer treated with third-generation chemotherapy regimens based on Eastern Cooperative Oncology Group data. J Clin Oncol 23:175–83. 15625371 10.1200/JCO.2005.04.177

[17] Arinc S, Ece F, Ertugrul M, Erdal N, Oruc O, et al (2009) Prognostic factors of elderly and young advanced stage NSCLC cases. South Med J 102 10:1019–22. 10.1097/SMJ.0b013e3181b66b11 19738522

[18] Subramanian J, Morgensztern D, Goodgame B, Baggstrom MQ, Gao F, et al (2010) Distinctive characteristics of non-small cell lung cancer (NSCLC) in the young: a surveillance, epidemiology, and end results (SEER) analysis. J Thorac Oncol 5:23–28. 19934774 10.1097/JTO.0b013e3181c41e8d

[19] Shigematsu H, Lin L, Takahashi T, Nomura M, Suzuki M, et al (2005) Clinical and biological features associated with epidermal growth factor receptor gene mutations in lung cancers. J Natl Cancer Inst 97:339–346. 15741570 10.1093/jnci/dji055

[20] Hirsch FR, Spreafico A, Novello S, Wood MD, Simms L, et al (2008) The prognostic and predictive role of histology in advanced non-small cell lung cancer: a literature review. J Thorac Oncol 3:1468–81. 19057275 10.1097/JTO.0b013e318189f551

[21] Forrest LM, McMillan DC, McArdle CS, Angerson WJ, Dagg K (2005) A prospective longitudinal study of performance status, an inflammation-based score (GPS) and survival in patients with inoperable non-small cell lung cancer. Br J Cancer 92(10):1834–6. 15870712 10.1038/sj.bjc.6602591 PMC2361776

[22] Stanley KE (1980) Prognostic factors for survival in patients with inoperable lung cancer. J Natl Cancer Inst 65:25–32. 6930515

[23] Rami Porta R, Crowley JJ, Goldstraw P (2009) The revised TNM staging system for lung cancer. Ann Thorac Cardiovasc Surg 15(1):4–9. 19262443

[24] Teramukai S, Kitano T, Kishida Y, Kawahara M, Kubota K, et al (2009) Pretreatment neutrophil count as an independent prognostic factor in advanced non-small-cell lung cancer: an analysis of Japan Multinational Trial Organisation LC00–03. Eur J Cancer 5:1950–8. 10.1016/j.ejca.2009.01.023 19231158

[25] Tibaldi C, Vasile E, Bernardini I, Orlandini C, Andreuccetti M, et al (2008) Baseline elevated leukocyte count in peripheral blood is associated with poor survival in patients with advanced non-small cell lung cancer: a prognostic model. J Cancer Res Clin Oncol 134:1143–9. 18347812 10.1007/s00432-008-0378-2 PMC12161754

[26] Mandrekar SJ, Schild SE, Hillman SL, Allen KL, Marks RS, et al (2006) A prognostic model for advanced stage non-small cell lung cancer. Pooled analysis of North Central Cancer Treatment Group trials. Cancer 107:781–92. 16847887 10.1002/cncr.22049

[27] Wigren T (1997) Confirmation of a prognostic index for patients with inoperable non-small cell lung cancer. Radiother Oncol 44:9–15. 9288851 10.1016/s0167-8140(97)00087-x

[28] Goldman RD, Kaplan NO, Hall TC (1964) Lactic dehydrogenase in human neoplastic tissues. Cancer Res 24:389–399. 14147812

[29] Koukourakis MI, Giatromanolaki A, Sivridis E, Bougioukas G, Didilis V, et al (2003) Lactate dehydrogenase-5 (LDH-5) overexpression in non-small-cell lung cancer tissues is linked to tumour hypoxia, angiogenic factor production and poor prognosis. Br J Cancer 89:877–885. 12942121 10.1038/sj.bjc.6601205 PMC2394471

[30] Danner BC, Didilis VN, Wiemeyer S, Stojanovic T, Kitz J, et al (2010) Long-term survival is linked to serum LDH and partly to tumour LDH-5 in NSCLC. Anticancer Res 30:1347–1351. 20530451

[31] Arrieta O, Michel Ortega RM, Villanueva-Rodriguez G, Serna-Thome MG, Flores-Estrada D, et al (2010) Association of nutritional status and serum albumin levels with development of toxicity in patients with advanced non-small cell lung cancer treated with paclitaxel-cisplatin chemotherapy: a prospective study. BMC Cancer 10:1471–1478. 10.1186/1471-2407-10-50 PMC2843671 20170547

[32] Kawai H, Ota H (2012) Low perioperative serum prealbumin predicts early recurrence after curative pulmonary resection for non-small-cell lung cancer. World J Surg 36:2853–7. 22948197 10.1007/s00268-012-1766-y

[33] Ferrigno D, Buccheri G (2003) Hematologic counts and clinical correlates in 1,201 newly diagnosed lung cancer patients. Monaldi Arch Chest Dis 59:193–198. 15065314

[34] Mundy GR (1988) Hypercalcemia of malignancy revisited. J Clin Invest 82:1–6. 3292583 10.1172/JCI113555 PMC303467

[35] Odell WD (1997) Endocrine/metabolic syndromes of cancer. Semin Oncol 24:299–317. 9208886

[36] Burdett S, Stephens R, Stewart L, Tierney J, Auperin A, et al (2008) NSCLC Collaborative Group. Chemotherapy in addition to supportive care improves survival in advanced non-small cell lung cancer: a systematic review and meta-analysis of individual patient from 16 randomized controlled trials. J Clin Oncol 26:4617–25. 18678835 10.1200/JCO.2008.17.7162 PMC2653127

